# Emergency Reversal of Anticoagulation

**DOI:** 10.5811/westjem.2018.5.38235

**Published:** 2019-08-06

**Authors:** Jennifer Yee, Colin G. Kaide

**Affiliations:** Wexner Medical Center, The Ohio State University, Department of Emergency Medicine, Columbus, Ohio

## Abstract

Owing to the propensity of anticoagulated patients to bleed, a strategy for reversal of anticoagulation induced by any of the common agents is essential. Many patients are anticoagulated with a variety of agents, including warfarin, low molecular weight heparin, and the direct oral anticoagulants such as factor Xa and factor IIa inhibitors. Patients may also be using antiplatelet agents. Recommendations to reverse bleeding in these patients are constantly evolving with the recent development of specific reversal agents. A working knowledge of hemostasis and the reversal of anticoagulation and antiplatelet drugs is required for every emergency department provider. This article reviews these topics and presents the currently recommended strategies for dealing with bleeding in the anticoagulated patient.

## INTRODUCTION

Inappropriate bleeding is the most concerning complication of anticoagulant therapy. The risk of bleeding varies with the type of anticoagulant agent used.^1^ The incidence of bleeding while on warfarin has been estimated at 15–20% per year, with life-threatening bleeding occurring at a rate of 1–3% per year.^2^ In 2010, atrial fibrillation alone prompted about 30 million prescriptions for warfarin.^2^ This does not include the many additional disease processes for which warfarin was indicated. In addition, the use of the direct oral anticoagulants (DOACs), such as factor Xa and factor IIa (thrombin) inhibitors, is rapidly increasing. Compared to warfarin, these drugs have generally been associated with lower rates of major hemorrhage and a reduction in the risk of fatal bleeding and intracranial hemorrhage (ICH).^3^ Owing to the propensity of anticoagulated patients to bleed, a strategy for reversal of anticoagulation induced by any of the common agents is essential for the treating clinician. We will review physiologic hemostasis processes, the effect of anticoagulation on normal hemostasis, and then discuss each anticoagulant and its reversal.

Providers should remember that all patients with emergent or life-threatening bleeding require attention to basic interventions, including cessation of anticoagulation therapy, blood product transfusions, and assessment for airway protection. Mechanical methods of hemostasis may be necessary, including direct compression, surgery, or embolization.

### Normal Hemostasis

Hemostasis occurs as part of a tightly regulated balance between clot formation and clot breakdown. Clot formation develops through an interaction of two independent processes—primary and secondary hemostasis. While the emergency physician does not need to have an intimate familiarity with all the details of the coagulation cascade, basic principles can guide the understanding of anticoagulants and reversal.

#### Primary Hemostasis

When damaged vascular endothelium is exposed, platelets bind with a glycoprotein binding complex (GPIIbIIIa) on the platelet and von Willebrand factor (vWF) on the endothelium. Platelets are then activated and release serotonin, platelet activating factor, platelet factor 4, thromboxane A2, and other substances, which attract, activate, and facilitate aggregation of other platelets.^4^ Primary hemostasis depends on platelet count and platelet function. Medications such as aspirin, nonsteroidal anti-inflammatory drugs, and others can inhibit platelet aggregation for varying durations. Platelet function testing reveals problems with platelet activity but is not done in real time so as to be useful in the emergency department (ED) setting.

#### Secondary Hemostasis

**Figure 1:**
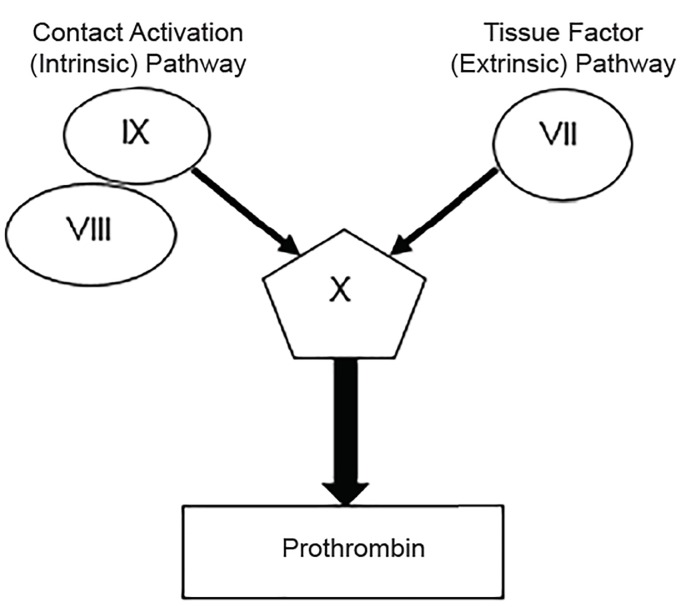
Parts of the coagulation cascade that are clinically relevant to the emergency physician.

This involves the generation of fibrin as a result of activation of the clotting cascade. Two pathways exist to initiate the cascade: the tissue factor (TF) pathway (formerly called the extrinsic pathway) and the contact activation pathway (formerly the intrinsic pathway) (Figure 1). The TF pathway is activated when an injury to the blood vessel allows factor VII (FVII) to come in contact with TF, which is expressed on stromal fibroblasts and leukocytes. The FVII-TF complex activates the common pathway leading to a large thrombin burst. This pathway is more clinically important as it generates the most fibrin in the shortest time. The contact activation pathway is initiated when collagen in the basement membrane of a blood vessel is exposed and a complex of high-molecular-weight kininogen (HMWK), prekallikrein, and FXII is formed. This causes the sequential activation of factors activating the common pathway culminating in fibrin formation. This pathway is less important in coagulation, but it plays a significant role in inflammation and innate immunity.

Fibrin crosslinks platelets, strengthening the primary platelet plug. For the system to function properly, there has to be an adequate quantity of functional clotting factors. Secondary hemostasis is tested by measuring the prothrombin time (PT) and the partial thromboplastin time (PTT) (Table 1).

### Impact of Anticoagulation Agents and Other Factors on Normal Hemostasis

**Figure 2:**
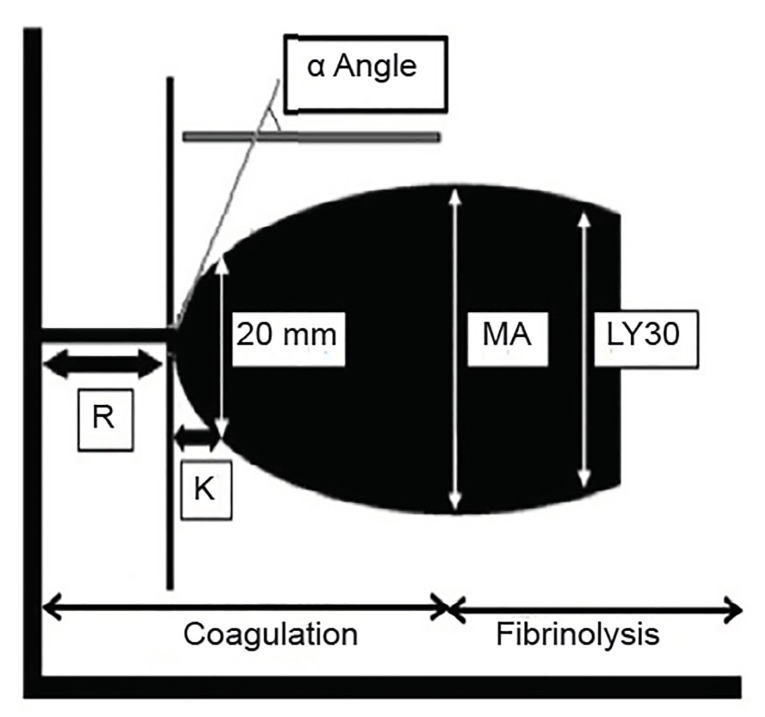
Thromboelastography (TEG)/rotational thromboelastometry graphic. *R*, reaction time, represents the time until initial fibrin formation. R reflects the coagulation factor levels present in the individual; *K*, coagulation time, from R until the amplitude of the TEG reaches 20 mm; *MA*, maximum amplitude, describes the maximum strength of the clot and reflects platelet function and fibrinogen activity; *α* angle, measures the speed of fibrin accumulation and cross linking and assesses the rate of clot formation; *LY30*, percentage diminution of the amplitude at 30 minutes after the maximum amplitude has been reached. LY30 represents a measure of the degree of fibrinolysis.^10^

Despite the complexity of the coagulation cascade, a basic familiarity with five coagulation factors (II, VII, VIII, IX, X) can explain almost all of the clinically relevant aspects of coagulation, anticoagulation and its reversal. For completeness, Factor VIII is included here because of its relevance to inherited clotting disorders: Factor VIII deficiency (hemophilia A) and Factor IX deficiency (hemophilia B). Patients with either of these diseases may present with bleeding (Table 1 and Figure 2). Commonly available tests include a PTT, PT, and international normalized ratio (INR) – a way of standardizing PT measurement across labs. Anti-Xa activity, thrombin time (TT), and ecarin clotting time (ECT) tests are often not readily available in the ED setting.

#### Activated Partial Thromboplastin Time (aPTT)

The aPTT is a measure of the contact activation (intrinsic) coagulation pathway; aPTT becomes prolonged in patients on heparin. It is not, however, a reliable measurement of anticoagulation in patients on low-molecular-weight heparin (LMWH) and with synthetic heparin chains such as fondadarinux (Arixtra) (a synthetic pentasaccharide). PTT will be prolonged in patients who are taking the factor II inhibitor dabigatran (Pradaxa). With increasing dabigatran plasma concentration, however, the response is curvilinear and flattens at higher dabigatran levels. These non-linear levels cannot be used to quantify effect. Therefore, the aPTT helps to identify that the patient has recently taken dabigatran but cannot assess the clinical degree of anticoagulation.^5^,^6^ A normal aPTT, in conjunction with a normal TT, excludes any clinically relevant anticoagulant activity of the drug.^7^

#### Prothrombin Time (PT)

PT and INR represent the changes to the TF (extrinsic) and common pathways. INR is prolonged with the use of warfarin. PT can also be prolonged with the use of rivaroxaban (Xarelto), an anti-Xa agent. The magnitude of PT/INR elevation, however, is not an effective measure of anticoagulation. PT/INR are very insensitive for detecting or predicting anticoagulation with the other anti-Xa agents apixaban (Eliquis) or edoxaban (Savaysa).^7^,^8^ Therapeutic dabigatran levels may slightly elevate the INR, but INR levels do not correlate with dabigatran activity.

#### Anti-Factor Xa Activity Assay

For these agents that primarily act on factor X, including the direct anti Xa agents, LMWH and fondaparinux, anti-Xa activity levels can be measured. Because it usually cannot be obtained in real time, the assay is rarely useful to make decisions in the ED setting.

#### Thrombin Time and Ecarin Clotting Time (ECT)

Thrombin clotting time directly assesses factor II activity by reflecting the conversion of fibrinogen to fibrin, while ECT assays test for factor II generation and has a strong linear correlation with the plasma concentrations of dabigatran. Both directly measure the activity of direct factor IIa inhibitors.^5^,^9^ Similar to the anti-factor Xa activity assay, these tests are not readily available or used in the clinical setting.

### Thromboelastography

**Figure 3:**
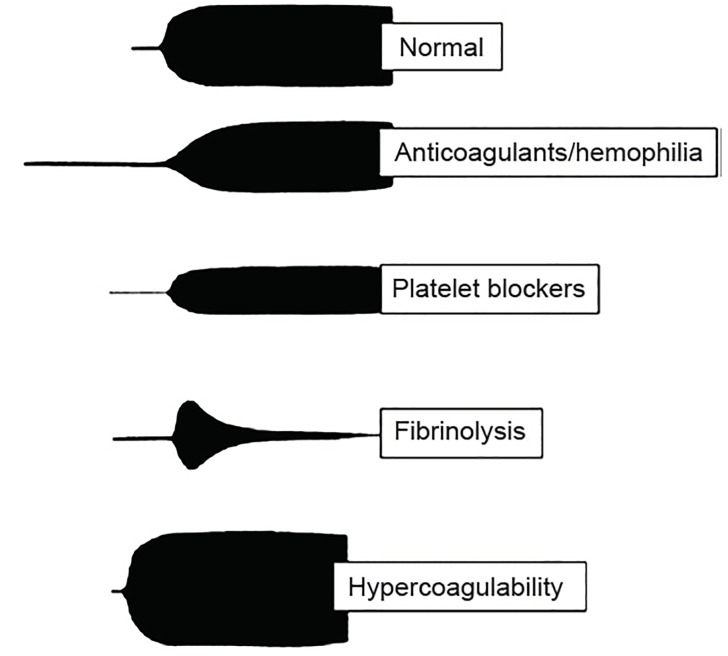
Interpretation of thromboelastography/rotational thromboelastometry graphics.^10^

Thromboelastography (TEG) and rotational thromboelastometry (ROTEM) are functional tests of coagulation that measure the interaction of clotting factors, fibrinogen, and platelets. The test determines the viscoelasticity of the clot during formation and breakdown. The whole blood sample is placed in a cup in which a pin is suspended from a torsion wire. The wire is connected to a mechanical-electrical transducer. As clotting progresses, increased tension in the coagulating blood alters the rotation detected by the pin. In TEG the cup is rotated, and in ROTEM the pin is rotated. These changes are converted into electrical signals, which then form a graphical representation (Figure 2). Measurements of the different phases of clotting and subsequent fibrinolysis are shown as changing of the shape of the graphic (Figure 3).^10^ Although TEG and ROTEM use slightly different nomenclature, the results are interchangeable.

TEG/ROTEM, in addition to the INR and PTT, can augment the understanding of the patient’s overall coagulation picture and help guide the need for transfusion of various blood products. There is growing interest in the use of TEG/ROTEM in trauma and other ED patients to assess the patient’s entire clotting process.^11^

### Reversal of Anticoagulation

#### Reversal of Warfarin

**Figure 4:**
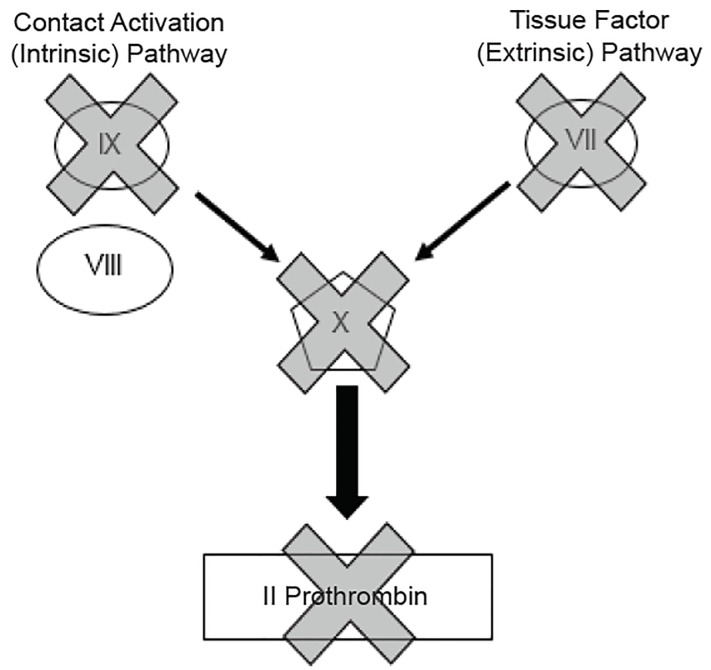
Where warfarin works.

Warfarin inhibits hepatic synthesis of vitamin K-dependent coagulation factors II, VII, IX, and X.^12^ This occurs through inhibition of vitamin K epoxide reductase and vitamin K1 reductase, which deplete vitamin HK2 (hydroquinone) and limit gamma-carboxylation of regulatory anticoagulant proteins C and S, as well as vitamin K-dependent coagulation (Figure 4).^13^

Vitamin K1 (phylloquinone) allows for the synthesis of vitamin K-dependent clotting factors de novo, while fresh frozen plasma (FFP) and prothrombin complex concentrates (PCCs) provide supplemental coagulation factors, including proteins C and S in some preparations. Vitamin K may be administered orally or intravenously. Due to erratic absorption, vitamin K should never be given via subcutaneous or intramuscular routes. Although the intravenous (IV) route has been associated with anaphylactoid reactions, the incidence of such reactions is extremely low (3/10,000).^15^ To further decrease the risk, it is advised to administer IV vitamin K over at least 20 minutes.^16^

When given intravenously, the INR begins to decrease within 1–2 hours^16^ and peaks in 4–6 hours.^17^ The 2012 American College of Chest Physician Guidelines recommend 10 milligrams (mg) of IV vitamin K for patients with life-threatening or emergent bleeding. See Table 2 for a summary of the 2012 Chest guidelines for reversal of Vitamin K antagonists.

FFP is derived from donor plasma that is rapidly frozen and stored at 18°C or colder.^16^ It contains all coagulation factors, as well as fibrinogen, protein C, and vWF. The intrinsic INR of FFP is 1.5, and it has not shown clinical benefit in patients with an INR below 1.7. Each unit of FFP has a volume of 200–250 milliliters (mL).^12^ Onset of action is 13–48 hours after administration. When FFP is ordered, it must undergo ABO blood group compatibility testing; Rh compatibility is not required. The plasma may take up to an hour to thaw, and then must be transfused urgently, as the labile clotting factors degrade with time.^16^ Human immunodeficiency virus and hepatitis transmission are known risks of transfusion, as well as the development of transfusion-related acute lung injury (TRALI) and allergic reactions.^16^

FFP is relatively cheap and widely available. However, administration is cumbersome. Dosing for life-threatening hemorrhage is 10–15 mL per kilogram of FFP, which averages to 4–5 units (800–1,250 mL) in an average-sized adult patient.^16^ FFP remains in the intravascular space and can precipitate fluid overload, and the evidence for its efficacy is only of low quality. Stanworth et al. noted that the reduction in INR was approximately 0.2 in 5000 FFP transfusions, performed for a broad range of indications.^18^

PCCs contain nonactivated coagulation factors II, VII, IX, and X, with varying amounts of proteins C and S. Both three- and four-factor concentrates contain these four factors. However, three-factor PCC contains lower (possibly negligible) amounts of factor VII.^16^ The concentrates are stored as a powder and may be reconstituted within minutes into a volume <100 mL.^12^ There are multiple dosing strategies, including a combination of INR and weight-based dosing, INR-based, and as a fixed dose. Onset of reversal occurs within 10–30 minutes, with an immediate decrease in INR to less than 1.5.^19^ Duration is 12–24 hours, and co-administration of vitamin K prevents rebound anticoagulation.^16^

PCC is administered as a small volume, has a quick onset, and results in immediate decrease in INR. The risks of TRALI and volume overload with FFP transfusions are eliminated. However, there is no significant evidence that PCC improves clinical outcomes or that it is superior to FFP, and it may be cost-prohibitive. A small risk of a prothrombotic state was established through a meta-analysis of 27 observational studies including 1032 patients. Twelve thromboembolic complications occurred (1.4%), two of which were fatal.^20^ A recent comparison of 4-factor PCCs to FFP has shown that the risk of inappropriate thrombosis is roughly the same.^21^

Recombinant factor VII, rVIIa (NovoSeven) is not recommended as a warfarin reversal agent.^22^,^23^,^24^ See Table 3 for a summary and dosing of reversal agents for warfarin.

#### Heparin Reversal

As reviewed by Hirsh and Raschke, unfractionated heparin binds to antithrombin through a high-affinity pentasaccharide.^25^ This complex then binds to factor II, irreversibly inhibiting factor II’s procoagulant activity, as well as coagulation factors Xa, IXa, XIa, and XIIa. The half-life of heparin is approximately 60 minutes.

**Figure 5:**
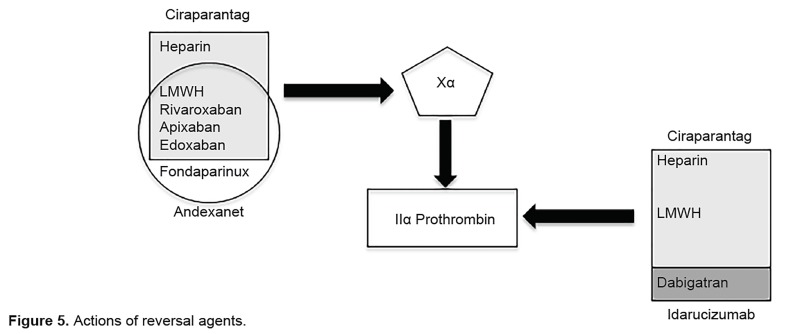
Actions of reversal agents.

Low-molecular-weight heparins (LMWH) are prepared by depolymerizing heparin. LMWH indirectly inhibits factor Xa activity by activating the antithrombin III complex, similar to heparin. This complex then inactivates factor Xa (Figure 5). These drugs also have a variable effect on factor II (prothrombin), with an anti-Xa to anti-II ratio that varies from 3:1 to greater than 5:1. The subcutaneous elimination half-life is 3–6 hours after injection and is not dose-dependent.^25^

Fondaparinux is a synthetic pentasaccharide that serves as a highly selective factor Xa inhibitor. It selectively binds to antithrombin III to inhibit factor Xa. Unlike heparin or LMWH, it does not inhibit factor II. There is rapid and complete bioavailability, and elimination half-life is 17–21 hours.^26^

Heparin is reversed by protamine, but protamine incompletely reverses factor Xa inhibition of LMWH despite complete neutralization of the antithrombin effect. This results in only about a 60% reversal of LMWH effects. If LMWH has been administered within the prior eight hours, 1 mg of protamine will neutralize 1 mg of enoxaparin.^27^ More than 50 mg of protamine will cause some anticoagulation by inhibition of factor V and is not recommended.

There is a paucity of human data on the reversal of fondaparinux. Human volunteer and animal studies suggest that recombinant activated factor VII may have some ability to partially normalize markers of anticoagulation in vivo.^28^ Activated PCC (aPCC), also known as “factor VIII inhibitor bypassing activity” (FEIBA), has been shown in animals to lessen bleeding and correct endogenous thrombin potential, which represents the amount of thrombin that can be generated after coagulation is activated by tissue factor in vitro.^29^ aPCC contains variable amounts of activated clotting factors with most of the activation occurring with factor VII.

Both andexanet alfa (a recombinant factor Xa)^30^ and ciraparantag^31^,^32^ (also known as aripazine) have been shown to bind to Xa inhibitors, but meaningful human studies on heparin, LMWH, and fondaparinux anticoagulated patients are lacking. See Table 4 for a summary of reversal agents for heparin, LMWH and fondaparinux.

### Direct Oral Anticoagulants (DOACs)

**Figure 6:**
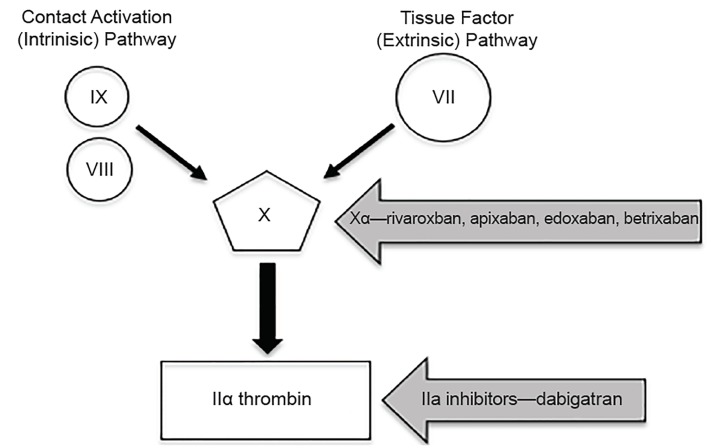
Where direct oral anticoagulants act.

DOACs are so named because they work by binding directly to factor Xa or factor II without the need to first complex with antithrombin (Figure 6). These agents were previously known as novel oral anticoagulants (NOACs), but the term “direct” is more appropriate. Two categories of agents are currently in use: direct factor IIa inhibitors (also called “direct thrombin inhibitors” or DTIs) such as dabigatran (Pradaxa) and the factor Xa inhibitors, including apixaban (Eliquis), rivaroxaban (Xarelto), edoxaban (Savaysa), and betrixaban (Bevyxxa).

Non-specific reversal agents in the form of 4-factor PCC (Kcentra in the United States) and aPCC (FEIBA) attempt to supplement the coagulation system with multiple clotting factors in hope of overwhelming the effect of the dabigatran. They are often considered when a specific reversal agent, idarucizumab (Praxbind) is not available. aPCC has been shown to reduce bleeding resulting from dabigatran in animal models^40^ and in healthy volunteers.^35^,^39^,^41^ Factor VIIa has shown mixed results in human volunteers.^42^, ^43^ In the absence of idarucizumab, FEIBA is the agent of choice when dabigatran reversal is needed.

#### A Specific Antidote

Idarucizumab (Praxbind) is a monoclonal antibody fragment that binds free and factor IIa-bound dabigatran. Dabigatran binds to idarucizumab with 350 times greater affinity than for factor II.^5^,^44^ It is the only U.S. Food and Drug Administration (FDA)-approved antidote for bleeding related to dabigatran. It is manufactured in 2.5 gram (g) vials, and it is administered as a 5 g total dose intravenously. See Table 5 for dosing of idarucizumab.

Pollack et al. reviewed the results of the prospective Reversal of the Anticoagulant Effects of Dabigatran by Intravenous Administration (RE-VERSE AD) clinical trial. Patients taking dabigatran who had serious bleeding or required urgent procedures were administered idarucizumab, and the results of the first 90 patients were reported. Of these patients with elevated clotting times at baseline, 88–98% had rapid and complete reversal of anticoagulant effects. One of 90 patients had a thrombotic event within 72 hours.^45^

In the 2017 follow-up study by Pollack et al, the full cohort of patients in the RE-VERSE AD clinical trial was analyzed. Two groups were studied. A 5 g dose of idarucizumab was administered to patients who received dabigatran therapy. Group A included 301 patients with life-threatening bleeding (98 patients with ICH and 137 with gastrointestinal [GI] bleeding). Group B included 202 non-bleeding patients requiring an urgent surgical procedure.^46^ The maximum percentage reversal of dabigatran was 100% (95% confidence interval, 100 to 100), as determined by diluted thrombin time (dTT) or ECT.^46^ ECT and dTT were chosen because they correlate linearly with dabigatran concentrations measured by mass spectroscopy. The article also reports a good correlation between these tests and the readily available aPTT.

In Group A, median time to cessation of bleeding among patients with ICH was 11.4 hours, and with GI bleeding was 3.5 hours. In Group B, the median time to procedure was 1.6 hours. The peri-procedural hemostasis was identified as normal by the treating clinician (using the International Society of Thrombosis and Haemostasis Bleeding Scale) in 188 patients of 202 patients (93%).^46^ At 30 days following idarucizumab administration, a total of 24 patients experienced a thrombotic event (4.8%), three of which were fatal. These included 12 venous thromboembolic events (VTE) including deep vein thrombosis (DVT) and/or pulmonary embolism (PE) or other systemic embolus, six myocardial infarctions, and six strokes. Of note, only 1.8% of the patients in this study were on dabigatran for VTE. The overall 30-day mortality rate was around 13%.^46^

Morbidity and mortality benefits of idarucizumab are unclear and are likely co-dependent on global management of these patients, including supportive care. Future rapid access to dabigatran concentrations may also guide reversal treatment and avoid unnecessary administration to those with low plasma drug levels.^47^

### Factor Xa Inhibitors: apixaban, rivaroxaban, edoxaban and betrixaban

Apixaban (Eliquis), rivaroxaban (Xarelto), edoxaban (Savaysa), and betrixaban (Bevyxxa) reversibly and competitively inhibit free and clot-bound factor Xa. With the exception of betrixaban, which has a more limited scope of indications, Xa agents are FDA-approved for stroke and systemic embolism prevention in patients with nonvalvular atrial fibrillation and for DVT and PE treatment. Rivaroxaban and apixaban are also approved for DVT prevention after hip or knee replacement surgery and to reduce the risk of recurrent DVT and PE. Betrixiban is approved for prophylaxis of VTE in hospitalized patients who are at significant risk for VTE.^48^–^50^ Median time to peak plasma concentration is approximately two hours for both apixaban and rivaroxaban, with steady-state concentrations reached by day four.^51^ The clinical effect of these drugs diminishes over time such that at 18 hours after the last dose, there is no indication for reversal.

Hemodialysis is not likely to be beneficial in cases of anticoagulation from apixaban or rivaroxaban since both of those drugs are more highly protein-bound. While edoxaban has relatively low protein binding, it is not well cleared by dialysis.^52^ Betrixaban is 60% protein bound, and it is not known if dialysis effectively clears the drug.^53^

#### Nonspecific Reversal Agents

The theory behind the use of these nonspecific reversal agents such as FEIBA, PCC, and rFVIIa is that they attempt to overwhelm the effect of a circulating Factor Xa inhibitor by supplementing either upstream factors (rVIIa) or factor X, along with both up and downstream factors. Patients taking Factor Xa inhibitors have normal levels of clotting factors and supplementation (in light of a circulating inhibitor) may not be effective, calling into question the potential efficacy of this strategy. Overall, there is limited patient data to support the use of nonspecific hemostatic agents for Factor Xa reversal, particularly with availability of a specific reversal agent.

Dzik (2015) reviewed the conflicting findings regarding factor Xa inhibitors and PCC use.^54^ Eerenberg et al (2011) studied healthy volunteers who took five doses of rivaroxaban over three days and subsequently received saline or a 4-factor PCC (Cofact). The PCC corrected the PT.^38^ Zahir et al. (2014) reviewed the effects of healthy volunteers who took a single dose of edoxaban and then took different doses of four-factor PCC (Beriplex). Laboratory testing and bleeding after a punch biopsy were then evaluated. Four-factor PCC reversed edoxaban’s effects on bleeding duration and endogenous thrombin potential, with complete reversal at 50 international units (IU)/kg. Effects on prothrombin time were partially reversed at 50 IU/kg.^55^ Levi et al. looked at healthy volunteers who took nine doses of rivaroxaban and then were randomly assigned to receive saline, 50 IU/kg 4-factor PCC (Beriplex), or 50 IU/kg of 3-factor PCC. The results showed that while 4-Factor PCC modestly and transiently reversed the PT, measured anti-Xa activity was identical after infusion of saline and 4-factor PCC.^56^

Multiple guidelines suggest PCC may be considered, but there are no definitive recommendations regarding its use.^57^–^60^ Turpie et al. (2012)^59^ and Spahn^58^ recommend 25–50 IU/kg, while Baumann Kreuziger et al. (2014) suggest 50 IU/kg of PCC.^60^ As described above, there is no consistent or significant evidence showing that PCC clinically reverses bleeding in real-world patients who are taking anti-Factor Xa anticoagulants. With the introduction of specific antidotes it is unlikely that PCC will remain a first-line reversal agent.^54^ Dosing of PCC and FEIBA for Factor Xa inhibitor reversal are listed in Table 6.

#### Specific Antidotes

Andexanet alfa, now officially known by the new generic name “coagulation factor Xa (recombinant), inactivated-zhzo,” and by the trade name Andexxa, is a specific factor Xa reversal agent. It was approved by the FDA in May 2018 and became commercially available in the first quarter of 2019. Andexanet alfa (we have chosen to use the more familiar and easier generic name) is a recombinant, modified factor Xa-like protein that acts as a “decoy molecule.” It binds factor Xa inhibitors with high affinity, yet owing to the designed lack of a membrane-binding carboxyglutamic acid (GLA) domain, it is functionally inactive and cannot participate in coagulation.^61^,^62^

The initial, healthy volunteer studies of this drug (Annexa-A and Annexa-R) showed rapid reduction of anti-factor Xa activity and restoration of thrombin generation in a total of 100 study subjects compared to 44 patients in the control groups. These patients were all anticoagulated with either rivaroxaban or apixaban and then given either a bolus only of andexanet or a bolus plus infusion. There were no thrombotic events or serious adverse events reported. Minor side effects occurred in 13 patients and were limited to dysgeusia (n = 2), feeling hot (n = 4), flushing (n = 6), and uncomplicated urticaria (n = 1).^30^

The phase 3b-4 study (ANNEXA-4) was published in February 2019. This manufacturer-sponsored study evaluated patients taking factor Xa inhibitors presenting with acute life-threatening or uncontrolled bleeding to assess reduction in anti-Xa activity as well as hemostasis and safety. The study, which included 352 patients with primarily intracranial (64%) or GI (26%) bleeding, showed that andexanet alfa rapidly reduced anti-Xa activity with effective hemostasis as judged by an independent committee using predetermined criteria adapted from those used in efficacy studies of 4-factor PCCs^63^ (Table 7). Safety was evaluated in all 352 patients while efficacy was evaluated in 254 patients.

Following administration of the andexanet alfa bolus, the median anti-Xa activity decreased by 92% among patients treated with rivaroxaban (n=100) and apixaban (n=134). This decrease was maintained during the two-hour infusion.^63^ Thrombin generation was restored to baseline in 100% of the patients. Hemostasis was evaluated at 12 hours and adjudicated as excellent or good in 82% of patients overall. Specifically, 85% of the GI bleeds and 80% of the intracranial hemorrhages had good or excellent hemostasis. This was well after termination of the two-hour infusion and during the time when the anticoagulant effects were beginning to return as measured by a rise in anti-Xa activity. As the process of forming clots is rapid, it is postulated that a stable clot was formed during the time of infusion when anticoagulation was reversed and this accounted for the hemostatic efficacy despite the short, two-hour duration of infusion. In no cases was the infusion continued for longer than two hours. Optimal dosing using a longer infusion is unclear and not addressed in ANNEXA-4. Further studies will be needed to see if the high cost of the drug can be offset by additional benefits to the patient. See Table 8 for cost of various reversal agents.

Thirty-four patients (10%) receiving andexanet had a thrombotic event by 30 days with 11 events occurring within five days after receiving andexanet. The remaining 23 patients had their thrombotic event during the time period of 6 and 30 days after treatment.^63^ These events included myocardial infarction, ischemic stroke of uncertain classification, transient ischemic attack, DVT and PE. Two important caveats should be noted regarding thromboembolic events: 1) Since most thrombotic events occurred after andexanet was cleared and no longer affecting hemostatic function, these thrombotic events are more likely secondary to underlying prothrombotic states for which patients were originally anticoagulated. In ANNEXA-4, significantly more (24%) of the patients enrolled were on anticoagulation for a thromboembolic event as compared to studies of PCCs for Xa inhibitor reversal and idarucizumab for dabigatran.^63^,^45^,^64^ 2) 26 of the 34 thromboembolic events occurred before the patients were restarted on anticoagulation, and only eight cases developed after anticoagulation was resumed.^63^ Our conclusion to this is that hypercoagulable patients have a higher propensity to clot when their anticoagulant is reversed and that the timely reinitiation of anticoagulant therapy is important to mitigate these thrombotic events.

ANNEXA-4 reported an all-cause, 30-day mortality rate of 14% (n = 49), of which 71% (n = 35) were cardiovascular in cause, 24% (n = 12) non-cardiovascular, and 5% (n = 2) of unknown etiology.^63^ The study was not designed to compare mortalities directly and they did not report any significance to the overall mortality and ICH mortality data. However, in studies comparing warfarin and rivaroxaban or apixaban for atrial fibrillation, historically the overall mortality is 20% with ICH mortality approaching 50%.^65^–^67^ Dosing of andexanet is shown in Table 9.

Ciraparantag, also known as aripazine or PER977, is a synthetic molecule that binds to unfractionated and LMWHs, as well as fondaparinux, dabigatran, and factor Xa inhibitors.^68^ It is thought to create a downstream procoagulant state.^69^ Ansell et al. performed a phase I clinical trial of healthy volunteers who were given a dose of edoxaban and then administered aripazine. Anticoagulation was reversed in 10 minutes as shown by decreased whole-blood clotting time, and effects lasted for 24 hours without procoagulant activity.^70^ Further human trials are needed to assess clinical outcomes and safety profiles. A direct comparison of the clinical efficacy of ciraparantag versus andexanet alfa has yet to be made. Sites of action of ciraparantag, andexanet, and idarucizumab are depicted in Figure 5.

### Reversal of Antiplatelet Agents

Aspirin irreversibly inhibits cyclooxygenase (COX)-1 and COX-2 enzymes to cause downstream inhibition of thromboxane A_2_, while thienopyridines such as clopidogrel (Plavix), ticlopidine (Ticlid), and prasugrel (Effient) irreversibly inhibit the P2Y12 receptor for adenosine diphosphate (ADP) on platelets, preventing ADP binding and platelet aggregation. Ticagrelor (Brilinta) and cangrelor (Kengreal) reversibly inhibit the ADP receptor, and dipyridamole (Persantine) reversibly inhibits ADP uptake by platelets. There is some controversy on how to manage patients on aspirin, clopidogrel, and other antiplatelet drugs. There are no guidelines for reversal of anti-platelet agents, but one in vitro model showed 2–3 units (4 or 6-packs) or 2–3 single-donor apheresis units of platelets added to plasma from healthy volunteers induced a normalization of platelet function.^71^

Gutermann et al. reviewed available guidelines related to antiplatelet therapy and gastrointestinal hemorrhage.^72^ There are no clear, clinical practice guidelines to dictate treatment of acute, life-threatening bleeding other than discontinuing anticoagulant and antiplatelet therapies. The Platelet Transfusions for Intracerebral Hemorrhage (PATCH) trial reported that platelet transfusion for spontaneous ICH in patients on antiplatelet therapy did not reduce bleeding and led to increased mortality and dependence at three months.^73^ Although frequently requested by surgical consultants, there is not enough evidence to make routine platelet transfusion a “standard of care.”^74^ Desmopressin, or DDAVP, increases endothelial release of vWF and factor VIII. It may be used to reverse the antiplatelet effects of aspirin and clopidogrel. DDAVP was evaluated by a meta-analysis in elective or emergent cardiac surgery in patients on antiplatelet therapy or had measured platelet dysfunction. Its use resulted in 25% less total volume of red blood cells transfused, 23% less blood loss, and a smaller risk of reoperation due to bleeding. There was no decrease in mortality or increase in thrombotic events, however, and DDAVP patients had an increase in clinically significant hypotension. The overall quality of evidence was judged to be low to moderate. Included trials were small, and five of the 10 trials were performed more than 20 years ago.^75^ Guidelines from the Neurocritical Care Society and Society of Critical Care Medicine support the use of a one-time 0.4 micrograms per kilogram IV dose of DDAVP in patients on antiplatelet therapy with ICH.^76^ Dosage of platelets and DDAVP for antiplatelet reversal are summarized in Table 10.

The reason patients are taking antiplatelet medications should be reviewed. Providers must assess the harm/benefit ratio of reversal, particularly in patients with recent coronary stent placement. In general, patients who received a bare metal stent are advised to stay on antiplatelet agents for one month. Those who received a drug-eluting stent should be on antiplatelet therapy for a minimum of six months, depending on the generation of stent (first or second).^77^ The main concern is that these patients have a higher risk of stent thrombosis if antiplatelet therapy is discontinued prematurely.

## DISCUSSION

Reversal of anticoagulation requires basic knowledge of underlying physiology of hemostasis, as well as obtaining a thorough history. A key piece of information is the timing of the last dose of anticoagulant agent. This is particularly important for DOAC agents where testing for degree of anticoagulation is not easily obtained or timely. Reversal agents for DOAC drugs are generally not indicated if the last known dose was greater than 18 hours prior to presentation. When real-time anti-Xa activity testing becomes widely available it will be very helpful in guiding the need for reversal when the last known dose is not available. If TEG/ROTEM is available, the results may likewise be helpful in this setting.

One of the most important overriding questions is this: “Does reversal of anticoagulation really have a clinically relevant benefit to the patient?” Most of the literature published on specific reversal agents such as 4-factor PCC, idarucizumab and andexanet focus on the agent’s ability to normalize tests of coagulation (INR, PTT, etc). Improvement in predetermined clinical markers of bleeding has been demonstrated by looking at the decrease in hematoma growth and limitation of a drop in hemoglobin. Finally, there are suggestions in the literature, mostly based on observation, that there appears to be less bleeding in patients for whom anticoagulation is reversed. A leap is then often made to imply that less bleeding directly translates into improved morbidity and/or mortality. *A morbidity or mortality benefit, however, has not yet been definitively demonstrated*. It is critical to determine if expensive reversal agents that may promote thrombosis are actually beneficial.

Randomized studies of a particular reversal agent vs placebo in bleeding patients will likely never be performed due to ethical concerns. Future studies should report individual patient data and describe detailed outcomes of patients receiving reversal agents, possibly comparing them to historical controls in the era prior to specific reversal agents. Future directions, including further evaluation of ciraparantag and andexanet alfa, especially regarding morbidity and mortality are hopefully in the pipeline. Additional research into the utility of TEG/ROTEM to guide transfusion of blood products and its effects on mortality are also warranted.

## CONCLUSION

Hemostasis is a complex, tightly regulated balance between bleeding and clotting. Through the use of anticoagulant agents, patients can be made to bleed, and with reversal agents (some of which are procoagulants by nature), patients can be forced to clot. In each of these situations, the harms and benefits should be weighed in the best interest of the patient and situation. Hopefully in the near future, safer anticoagulants and more-specific reversal agents will become available, along with easy access to specific testing that can guide our use of these powerful medications.

## Figures and Tables

**Table 1:** Laboratory testing of hemostasis.

Test	Range	Components Tested	Medications
Prothrombin Time (PT/INR)	12–13 sec/0.8–1.2	Tissue factor pathway and common pathway (II, VII, X)	Warfarin, anti-Xa agents (rivaroxaban*, apixaban*, edoxaban*)
Partial Thromboplastin Time (PTT)	30–60 seconds	Contact activation and common pathways (all factors except factor VII)	Heparin, factor II inhibitors (dabigatran**)
Anti-Xa Assay	0.0	Factor X	LMWH, anti-Xa agents (rivaroxaban*, apixaban*, edoxaban), fondaparinux
Thrombin Time	12–14 seconds	Factor II activity	Factor IIa inhibitors (dabigatran)
Ecarin Clotting Time (ECT)	22.6 to 29.0 seconds At trough: >3x the upper limit of normal suggests bleeding risk	Factor II activity	Factor IIa inhibitors (dabigatran)

*PT/INR*, prothrombin time/international normalized ratio; *LMWH*, low-molecular-weight heparin.

* PT is frequently elevated with these agents but a prediction as to the degree of anticoagulation is unreliable with these agents.

** PTT is useful in determining the presence of an anti-factor II activity, however it cannot be used to monitor the degree of anticoagulation produced by these medications.

**Table 2:** Recommendations for managing increased international normalized ratios or bleeding in patients rreceiving Vitamin K antagonists.

Condition	Description
INR above therapeutic range but <5.0; no significant bleeding	Lower dose or omit dose, monitor more frequently, and resume at lower dose when INR therapeutic; if only minimally above therapeutic range, no dose reduction may be required.
INR ≥5.0 but ≤10.0; no significant bleeding	Omit next one or two doses, monitor more frequently, and resume at lower dose when INR in therapeutic range. Alternatively, omit dose and give vitamin K1 (1–2.5 mg orally), particularly if at increased risk of bleeding. If more rapid reversal is required because the patient requires urgent surgery, vitamin K1 (2–4 mg orally) can be given with the expectation that the INR will decrease in 24 hours. If the INR is still high, additional vitamin K1 (1–2 mg orally) can be given.
INR >10.0; no significant bleeding	Hold warfarin therapy and give higher dose of vitamin K1 (5–10 mg orally) with the expectation that the INR will be reduced substantially in 24–48 hours. Monitor more frequently, and use additional vitamin K1 if necessary. Resume therapy at lower dose when INR therapeutic.
Serious or life-threatening bleeding at any elevation of INR	Hold warfarin therapy and give vitamin K1 (10 mg by slow IV infusion), supplemented with 4-factor prothrombin complex concentrate or fresh frozen plasma. Vitamin K1 can be repeated every 12 hours.

* Adapted from Holbrook A, et al. Evidence-Based Management of Anticoagulant Therapy: Antithrombotic Therapy and Prevention of Thrombosis, 9th ed: American College of Chest Physicians Evidence-Based Clinical Practice Guidelines. *Chest*. 2012;141:e152S–184S.

**Table 3:** Summary and dosage of reversal agents for warfarin in life-threatening bleeding.

Agent	Dose	Additional Information
Vitamin K	1–10 mg IV	SC delivery is no longer used
PCC 3-Factor (Profilnine) 4 factor (Kcentra*)	Strategy 1: INR and Weight-Based Dosing INR 2–4: 25 IU/kg by IV push INR ≥4–6: 35 IU/kg by IV push INR >6: 50 IU/kg by IV push Strategy 2: INR-Based Dosing INR <5: 500 units; INR ≥5: 1000 units Strategy 3: Fixed Dose 1500 IU	INR-based dosing is most effective with 3-factor preparations. Absolute dosing strategies should not be used with 3-factor PCCs. Any of the 3 strategies can be used with 4-factor PCCs.

*mg*, milligram; *SC*, subcutaneous; *PCC*, prothrombin complex concentrate; *INR*, international normalized ratio; *IU*, international unit; *IV*, intravenous; *kg*, kilogram.

* U.S. Food and Drug Administration-approved for the reversal of warfarin-related bleeding.

**Table 4:** Dosage of reversal agents for heparin, low-molecular-weight heparins, and synthetic pentasaccharides—fondaparinux (Arixtra).

Agent	Dose	Additional Information
Protamine for Heparin	Time elapsed from last heparin dose: Dose of protamine (mg) to neutralize 100 units of heparin Immediate: 1–1.5 mg/100 units heparin 30–60 min: 0.5–0.75 mg/100 units heparin >2 h: 0.25–0.375/100 Units Heparin	Doses should not exceed 50 mg at a time.
Protamine for LMWH	Dalteparin (Fragmin): 1 mg protamine neutralizes 100 units dalteparin If bleeding continues or PTT remains prolonged 2–4 hours after protamine, may give a second protamine dose of 0.5 mg per 100 units dalteparin. Enoxaparin (Lovenox): if < 8 hours after last dose enoxaparin, give 1 mg protamine per 1 mg enoxaparin; If 8–12 hours after last dose enoxaparin, give 0.5 mg protamine per 1 mg enoxaparin. If >12 hours after last dose of enoxaparin (when enoxaparin administered q12h), protamine not required. If bleeding continues or PTT remains prolonged 2–4 hours after protamine, may give a second protamine dose of 0.5 mg per 1 mg enoxaparin.	Protamine may have some effect on LMWH. Only 60–75% of the anti Xa activity of LMWH is neutralized by protamine. Effectiveness depends on which LMWH is used. There is a real concern when using protamine with LMWH: Protamine when given by itself has anticoagulant effects. If there is reversal of the non-Xa activity and only partial (but not enough) reversal of the Xa activity, the net vector will point to anticoagulation. DO NOT EXCEED 50 mg per dose. Protamine only partially neutralizes anti-factor Xa activity (~60%). Fondaparinux: Has only anti-Xa activity and protamine will have no significant effect.
Reversal of Fondaparinux	Recombinant activated factor VII (NovoSeven): 90 mcg/kg IV Activated prothrombin complex concentrate (aPCC) FEIBA 50 U/kg IV	Very limited data to recommend these agents to reverse fondaparinux.

*mg*, milligram; *IV*, intravenous; *LMWH*, low-molecular-weight heparin; *PTT*, partial thromboplastin time; *q12h*, every 12 hours; *kg*, kilogram; *FEIBA*, factor VIII inhibitor bypassing activity.

**Table 5:** Dosage of reversal agents for dabigatran.

Agent	Dose	Additional Information
aPCC (FEIBA)	50 U/kg	May be more thrombogenic than non-activated PCC.
Antibodies to dabigatran (Idarucizumab)	5 g provided as two separate vials each containing 2.5 g/50 mL.	The only FDA-approved “antidote” to dabigatran-related bleeding.
Cryoprecipitate	2 bags	If fibrinogen is < 200 mg/dL, give 2 bags cryoprecipitate.

*aPCC*, activated prothrombin complex concentrate; *FEIBA*, factor VIII inhibitor bypassing activity; *U/kg*, units per kilogram; *g*, gram; *mL*, milliliter; *FDA*, U.S. Food and Drug Administration; *mg/dL*, milligrams per deciliter.

**Table 6:** Dosage of nonspecific reversal agents for anti factor Xα anticoagulants (rivaroxaban, apixaban, edoxaban, betrixaban).

Agent	Dose	Additional Information
4-Factor PCC (Kcentra)	25–50 units/kg	Not to exceed 5000 units. Repeat dosing is not recommended. This is generally considered the preferred agent for reversing anti-Xa Inhibitors.
aPCC (FEIBA)	25 units/kg	If still clinically significant bleeding, consider re-dosing, but no sooner than 6 hours.

*aPCC*, activated prothrombin complex concentrate; *FEIBA*, factor VIII inhibitor bypassing activity; *U/kg*, units per kilogram.

**Table 7:** Criteria for determining hemostatic efficacy in patients receiving andexanet.

For intracranial hemorrhage	Slowing in growth of hematoma size at one hour and 12 hours compared to baseline.
For gastrointestinal bleeding	A drop in hemoglobin of less than 10% from baseline at 12 hours was considered good hemostasis.
For visible bleeding	Cessation of bleeding at one-hour post andexanet was considered good hemostasis if bleeding stopped at 4 hours and no additional therapy was required.
For musculoskeletal b leeding	Decrease in pain, no objective signs of ongoing bleeding and absence of further swelling.

**Table 8:** Cost of reversal agents–based on an 80-kilogram patient.

Generic Drug	Trade Name	Dose	Approximate Cost
Phytonadione	Vitamin K	10 mg IV	$395.00^A^
FFP	N/A	4 units is usual minimum	$1000^B^ ($250 each)
4-Factor PCC	Kcentra	25–50 units/kg	$2,540 to $5,080^B^
Activated PCC	FEIBA	25 units/kg	$5,400^B^
Idarucizumab	Praxbind	5 grams	$3,600^C^
Andexanet (Low Dose)	Andexxa	400 mg bolus + 480 mg infusion	$24,750**
Andexanet (High Dose)*	Andexxa	800 mg bolus + 960 mg infusion	$49,500

*PCC*, prothrombin complex concentrate; *FEIBA*, factor VIII inhibitor bypassing activity; *IV*, intravenous; *units/kg*; units per kilogram; *mg*, miligram.

* High dose rarely used in Annexa-4 study protocol. Predicted to be rarely used in real-life practice.

** New technology add-on payment (NTAP) is available with the maximum NTAP reimbursement of $14,062.50, or 50% of the wholesale acquisition cost of the low dose. NTAP is expected to remain in effect for a period of 2–3 years, until the cost of andexanet alfa is included in the recalibration of the diagnosis related group payment rates.

A Phytonadione. https://www.drugs.com/price-guide/phytonadione. 2018.

B Wexner Medical Center at The Ohio State University pharmacy data. 2019.

C Praxbind. http://www.drugs.com/price-guide/praxbind. 2018.

**Table 9:** Dosing of andexanet.

Drug	Anti-Xα Dose	Time Since Last Dose	
		<8 Hours or Unknown	≥ 8 Hours
		
Rivaroxaban	≤ 10 mg	Low Dose	Low Dose
	> 10 mg or Unknown	High Dose	
Apixaban	≤ 5 mg	Low Dose	Low Dose
	> 5 mg or Unknown	High Dose	

*Low dose*, 400 milligrams (mg) at 30 mg/min followed by 4 mg/min for up to 120 min; *high dose*, 800 mg over 30 mg/min followed by 8 mg/min (milligrams per minute) for up to 120 min.

**Table 10:** Summary and dosage of reversal agents for platelet inhibitors—aspirin, clopidogrel, prasugrel, ticagrelor and nonsteroidal anti-inflammatory drugs.

Agent	Dose	Additional Information
Platelet transfusion	2–3 U of pooled platelets or 2–3 Apheresis U	Human studies proving the efficacy of the use of platelets in patients with anti-platelet agent induced bleeding are lacking.
DDAVP (Desmopressin)	0.4 μg/kg IV	Promotes platelet adherence. Consider for bleeding with platelet inhibitor use along with platelet transfusion.

*U*, unit; *μg/kg IV*, micrograms per kilogram intravenously.
